# Diabetic Kidney Disease‐Associated Pathological Angiogenesis: The Role of Aquaporin‐1

**DOI:** 10.1111/1753-0407.70159

**Published:** 2025-10-17

**Authors:** Fengyi Zhang, Jiayi Zhang, Xin Wang, Yaxin Chen, Ziyang Cheng, Jingjing Pan, Yufeng Zhang, Yujie Li, Wenbo Wang, Linhua Zhao

**Affiliations:** ^1^ First Clinical College of Shandong University of Traditional Chinese Medicine Jinan Shandong Province China; ^2^ Geriatrics Department, Second Affiliated Hospital of Shandong University of Traditional Chinese Medicine Jinan Shandong Province China; ^3^ Orthopedics Department, Second Affiliated Hospital of Shandong University of Traditional Chinese Medicine Jinan Shandong Province China; ^4^ Institute of Metabolic Diseases, Guang’anmen Hospital, China Academy of Chinese Medical Sciences Beijing China

**Keywords:** angiogenesis, Aquaporin‐1, diabetic kidney disease, review

## Abstract

Diabetic kidney disease (DKD) is recognized as one of the leading causes of chronic kidney disease (CKD) and end‐stage kidney disease (ESKD) worldwide, representing a rapidly growing global public health concern. Despite significant advances in understanding the complex pathophysiological mechanisms of DKD, curative treatments are currently unavailable, and the reversal of established renal injury remains an elusive goal in clinical practice. Among various pathological features, aberrant angiogenesis has been closely associated with glomerular injury and the early development of proteinuria in DKD, playing a crucial role in driving disease progression. However, the molecular mechanisms underlying this pathological angiogenesis in DKD remain incompletely understood and warrant further elucidation. Recent research has increasingly implicated aquaporins (AQPs), a family of transmembrane water channel proteins, in the pathogenesis of both acute and chronic kidney disorders, including DKD. In particular, aquaporin‐1 (AQP1), which is highly expressed in renal tissues, has emerged as a potential modulator of angiogenic activity within the kidney microenvironment. Although AQP1 and aberrant angiogenesis have been individually explored in the context of DKD, no comprehensive review has systematically examined their interrelationship. This review consolidates current evidence regarding the role of AQP1 in pathological angiogenesis during DKD progression, highlighting its potential significance and identifying gaps that warrant further investigation.

## Graphical abstract

Hyperglycemia upregulates AQP1 expression in renal endothelial cells, promoting abnormal angiogenesis. Structurally and functionally immature vessels lead to glomerular capillary leakage, proteinuria, and progressive renal injury. Targeting AQP1 may attenuate abnormal angiogenesis in DKD and slow disease progression.

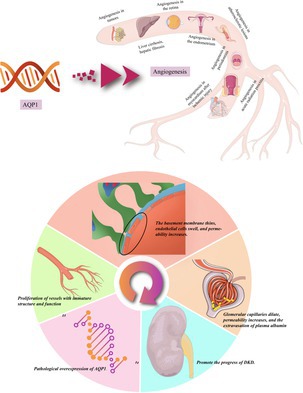

**Summary**

- This article provides a detailed review and summary of the potential association between AQP1 and the progression of DKD.
- AQP1 may serve not only as a biomarker of vascular remodeling in DKD, but also as a key mediator contributing to disease progression.

## 1 Introduction

In 2021, diabetes mellitus affected approximately 10.5% of the global population aged between 20 and 79 years, corresponding to roughly 536.6 million individuals worldwide. This number is projected to further increase, reaching an estimated 783.2 million people (12.2%) by 2045, thus reflecting a significant and ongoing global health challenge [1]. Among the diabetic population, it is estimated that up to 35% of individuals will develop some form of kidney involvement during the course of their disease [2]. DKD has emerged as one of the leading causes of chronic kidney disease (CKD) and end‐stage kidney disease (ESKD), conditions that often necessitate renal replacement therapies such as dialysis or kidney transplantation [3, 4]. A major clinical manifestation of DKD is diabetic nephropathy (DN), which is characterized by persistent proteinuria and a progressive decline in renal function. DN affects approximately 30%–40% of diabetic patients over their lifetime, contributing substantially to morbidity and mortality [5, 6]. Besides DN, DKD also encompasses non‐proteinuric phenotypes including renal arteriosclerosis, arteriolosclerosis, and episodes of acute kidney injury associated with cardiovascular events [7]. As diabetes increasingly affects younger individuals with fewer comorbidities and longer lifespans, the epidemiology of DKD is undergoing a shift where prevalence is rapidly surpassing incidence [5]. Complications related to DKD, including cardiovascular disease, stroke, and hypertension, impose substantial healthcare costs owing to frequent hospitalizations, long‐term medication requirements, and extensive outpatient care [8].

DKD remains largely irreversible once structural renal damage occurs [9]. Although recent pharmacological advances have led to the development of therapies such as sodium‐glucose cotransporter‐2 (SGLT2) inhibitors and renin‐angiotensin‐aldosterone system (RAAS) blockers, as well as novel agents targeting pathological signaling pathways including protein kinase C (PKC), transforming growth factor‐beta (TGF‐β), Janus kinase/signal transducers and activators of transcription (JAK/STAT), and oxidative stress, their clinical efficacy remains limited by incomplete mechanistic understanding, suboptimal utilization, and challenges in translation [10, 11]. Consequently, lifestyle interventions, including smoking cessation, weight management, blood pressure control, and optimal glycemic regulation, remain fundamental components of DKD prevention and management strategies [12, 13].

**FIGURE 1:**
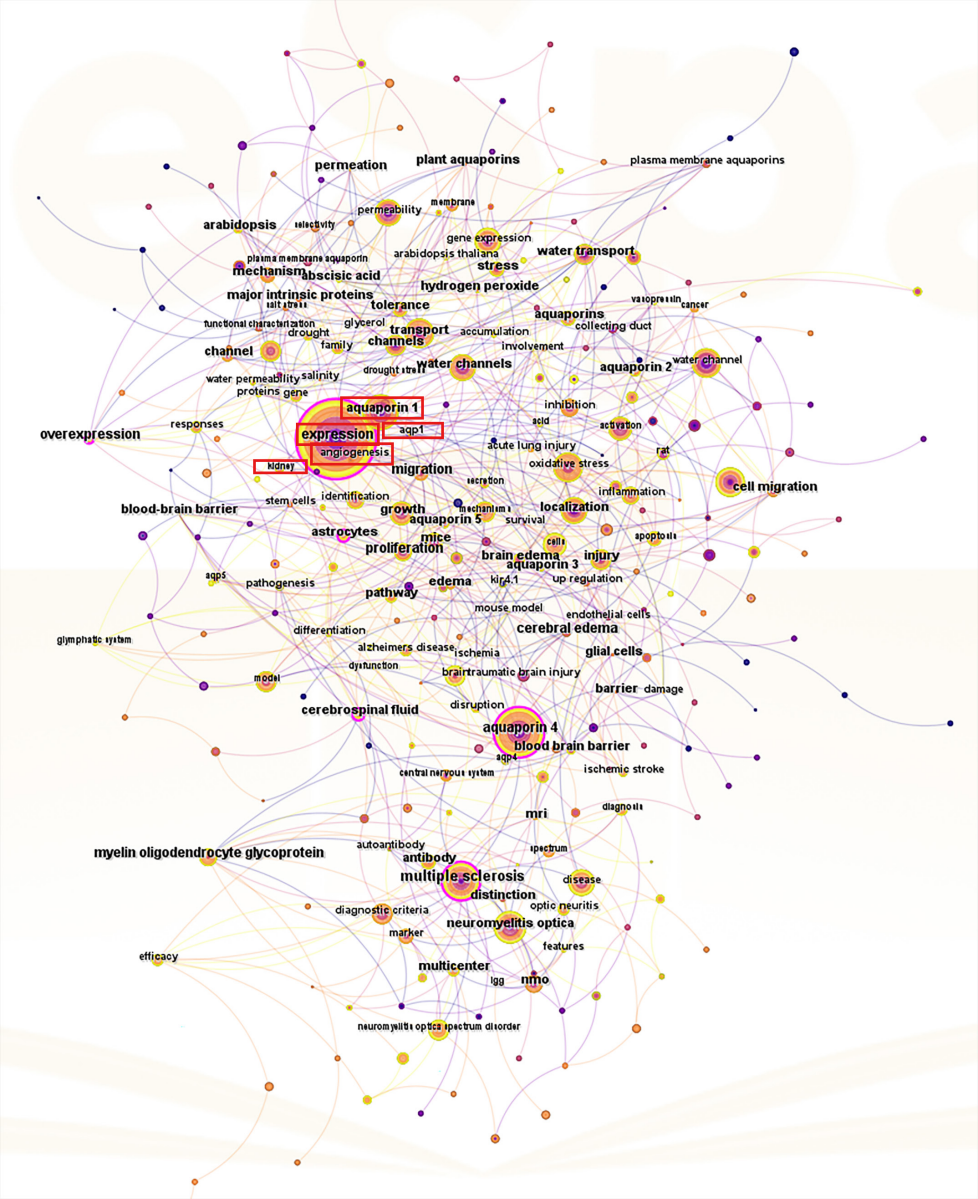
Keyword co‐occurrence map of AQP1‐related publications (2015–2024, Web of Science Core Collection). The visualization was generated using CiteSpace, illustrating the interconnection between AQP1, angiogenesis, and renal‐related topics.

In recent years, increasing attention has been focused on the role of AQPs in the pathophysiology of renal and systemic diseases. AQPs are involved in water transport and have been implicated in various kidney conditions, ranging from acute kidney injury to chronic kidney diseases such as DKD [14]. Among the AQP family, AQP1 is the most widely expressed isoform in the kidney; however, its specific role in DKD has yet to be fully characterized. Bibliometric analyses (Figure 1) conducted using CiteSpace (version 6.3.R1) on the literature published between 2015 and 2024 reveal that keywords including “angiogenesis,” “kidney,” and “expression” frequently co‐occur with AQP1‐related research, underscoring the emerging scientific interest in the intersection of these topics. Given the critical role of pathological processes like angiogenesis in DKD progression, understanding the specific contributions of molecules such as AQP1 within the kidney context is of significant interest.

## 2 Overview of AQP1

AQPs are a family of highly selective transmembrane channel proteins primarily responsible for the transport of water across cellular membranes, with certain members also mediating the transport of small molecular solutes [14]. They are ubiquitously expressed in a broad range of tissues, with predominant localization in epithelial and endothelial cell types, and are additionally found in erythrocytes, astrocytes, adipocytes, and skeletal muscle cells [15]. Dysregulation of AQP expression and function has been implicated in the pathogenesis and progression of numerous diseases, including various renal disorders, through mechanisms involving impaired water balance, oxidative stress, aberrant cellular signaling pathways, angiogenesis, and tissue remodeling [16, 17, 18, 19, 20].

**FIGURE 2:**
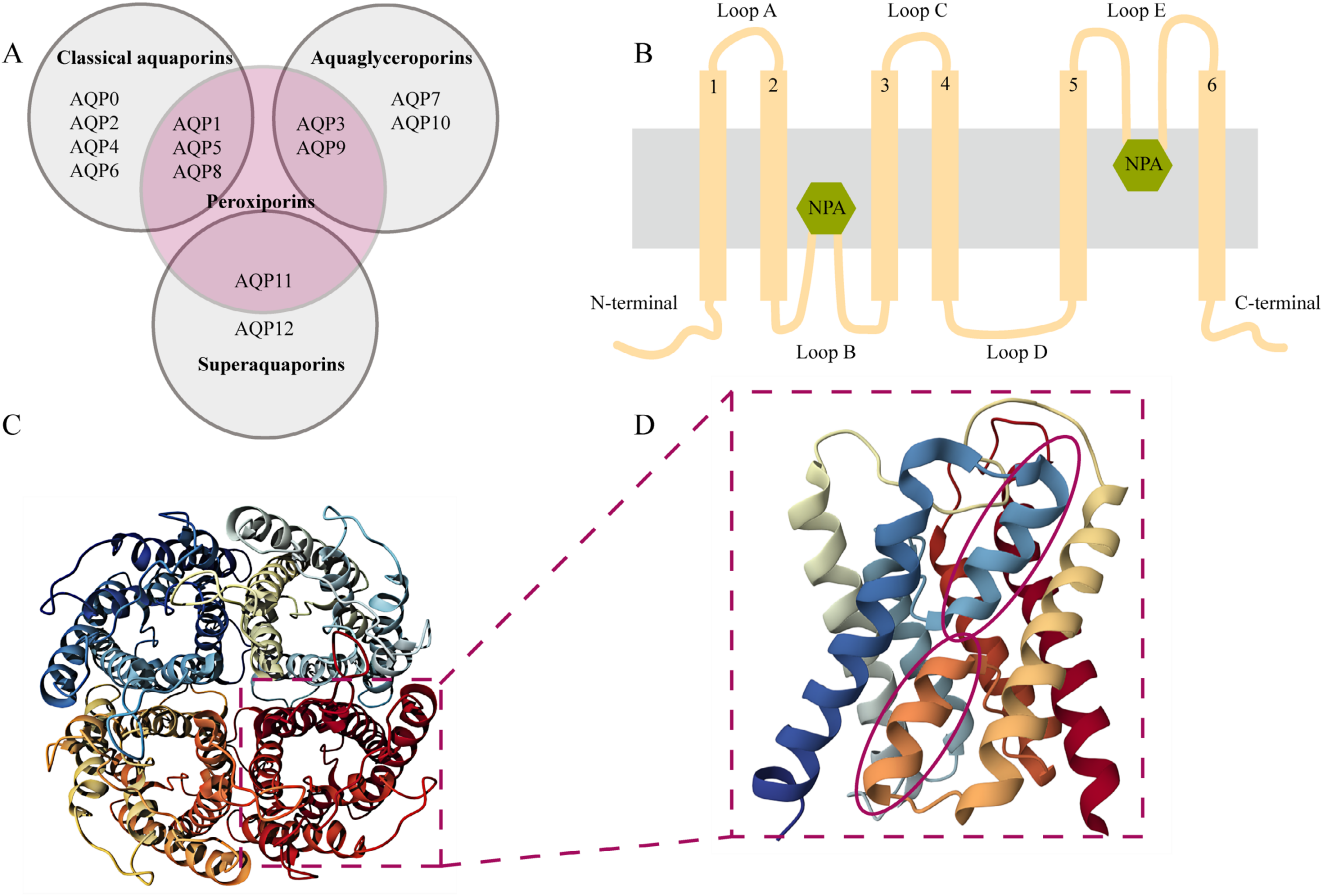
Classification and structural features of AQPs. (A) Classification of 13 AQP subtypes based on their primary structure and selectivity. (B) Schematic representation of AQP membrane topology, showing that each monomer consists of six transmembrane α‐helices [1, 2, 3, 4, 5, 6] connected by five loops (A–E), with conserved Asn‐Pro‐Ala (NPA) motifs embedded within the membrane. In a functional monomer, the hydrophilic loops B and E fold into the cavity formed by two half‐helices, bringing them into close spatial proximity to form a charge‐selective gating region containing two NPA motifs. (C) Top view of the homotetrameric structure of AQP1. (D) Side view of a single monomer, highlighting the two NPA motifs (circled in red).

Structurally, each AQP monomer is an approximately 28 kDa protein composed of six highly hydrophobic transmembrane α‐helical domains that collectively form a narrow, water‐selective pore [21] (Figure 2B,D). Functionally, these monomers operate as independent water channels, yet in vivo, four monomers oligomerize to assemble into a homotetrameric quaternary structure [22, 23, 24] (Figure 2C). The central pore formed by the tetrameric arrangement is predominantly hydrophobic, though its precise function remains unclear [25]. The human genome encodes 13 AQP isoforms (AQP0–AQP12), which are broadly classified into three principal subfamilies based on their sequence homology and substrate selectivity [26] (Figure 2A): [1] orthodox or classical water channels (AQP0, 1, 2, 4, 5, 6, 8), which primarily facilitate water transport, although AQP6 and AQP8 also mediate anion and ammonia transport, respectively; [2] aquaglyceroporins (AQP3, 7, 9, 10), which transport water as well as small uncharged solutes such as urea and glycerol; and [3] superaquaporins, also known as unorthodox AQPs (AQP11 and 12), which differ from other AQPs due to their unique evolutionary trajectory, reduced sequence similarity at the primary structure level, distinctive transport characteristics, and predominant intracellular localization [20, 27, 28, 29, 30, 31]. Recent research has further identified that several AQPs, including AQP1, 3, 5, 8, 9, and 11, exhibit permeability to hydrogen peroxide (H₂O₂), leading to the designation of a fourth subclass termed “peroxiporins” due to their role in modulating redox signaling and oxidative stress responses [32, 33, 34, 35, 36, 37].

AQP1, the first member of the AQP family to be identified [38], is widely expressed in microvascular endothelial cells beyond the central nervous system, including the renal microvasculature, as well as non‐vascular endothelia in tissues such as the pleura, peritoneum, cornea, and lymphatics [20, 39]. Similar to the basic structure of AQPs, AQP1 is a tetramer composed of four monomers, each containing six transmembrane α‐helices and a water‐selective pore that forms a characteristic hourglass‐shaped structure. These monomers are arranged in a right‐handed twist around a central pore, and this hourglass architecture is critical for AQP1's high permeability and strict water selectivity [40]. Additionally, the central pore formed by the AQP1 tetramer has been reported to facilitate the transport of ions and small molecules, such as potassium ions, oxygen, and ammonia [41, 42]. Functional knockout studies in murine models have demonstrated that loss of AQP1 disrupts physiological fluid regulation, inflammatory responses, cell migration, and angiogenesis, resulting in pathological alterations across multiple organ systems43. Collectively, these findings underscore the vital physiological role of AQP1 and highlight its potential as a therapeutic target in diverse human diseases [43].

## 3 Angiogenesis in DKD

Blood vessels, which are among the earliest functional structures formed during embryogenesis, constitute the largest network in the body. When dysregulated, they contribute to a broad spectrum of diseases, including malignancies, ischemia, inflammation, infections, and autoimmune disorders [44]. Pathological angiogenesis is recognized as a hallmark of various diseases [45, 46].

DKD, a prevalent microvascular complication of diabetes mellitus [47], is increasingly recognized to involve aberrant vascular proliferation contributing to its pathophysiology, though the precise mechanisms remain incompletely elucidated [48]. The earliest observations of abnormal glomerular vascular proliferation in diabetes date back to 1987 when Osterby and Nyberg first documented pathological angiogenesis within the glomeruli of patients with type 1 diabetes [49]. Subsequent studies in experimental models, such as streptozotocin‐induced diabetic rats, have consistently demonstrated comparable vascular remodeling characterized by glomerular enlargement attributable to neovascularization within the glomerular capillary network [50, 51]. Histopathological evidence indicates that neovascularization at the glomerular vascular pole, although occasionally present in normal kidneys, occurs significantly more frequently in diabetic nephropathy and correlates with albuminuria and severity of glomerular lesions [52]. Kanesaki et al. documented an increased number of glomerular vessels in patients with type 2 diabetic nephropathy, with neovascularization significantly correlated with vascular endothelial growth factor (VEGF) mRNA expression and mesangial matrix expansion [53]. Studies have demonstrated that hyperglycemia‐induced secretion of cytokines by mesangial cells promotes aberrant angiogenesis within the kidney [54]. Single‐nucleus RNA sequencing (snRNA‐seq) analyses of cryopreserved human diabetic kidney samples revealed pronounced vascular neogenesis features across glomerular cell types, proximal and distal tubules, and principal cells [55]. Moreover, mesenchymal stromal cells (MSCs) derived from patients with DKD exhibit transcriptional alterations associated with angiogenesis compared to age‐matched controls [56]. Collectively, angiogenesis may play a significant role in the pathogenesis of DKD [57, 58], and therapeutic modulation of angiogenic activity holds potential to mitigate progression to end‐stage renal disease in DKD patients [56].

## 4 AQP1 And Angiogenesis

AQP1 has been extensively studied for its involvement in pathological angiogenesis across a wide range of disease conditions, including malignant tumors, liver cirrhosis, atherosclerosis, diabetic retinopathy, and endometriosis. In oncological contexts, AQP1 expression is markedly upregulated within tumor microvasculature and has been demonstrated to facilitate tumor cell proliferation, migration, and angiogenesis. Functional ablation of AQP1, either via genetic knockout models or targeted gene silencing, results in a significant reduction in microvessel density and impaired expression of canonical angiogenic markers, underscoring its pivotal role in tumor neovascularization [59, 60, 61]. In liver pathology, elevated AQP1 expression has been documented in the neovascular networks associated with hepatic fibrosis and cirrhosis, suggesting a contributory role in promoting aberrant angiogenesis that exacerbates fibrotic progression [62]. Similarly, AQP1 knockout mouse models and in vitro studies using human retinal endothelial cells have provided compelling evidence for AQP1's critical function in ocular angiogenesis, a process central to the development of diabetic retinopathy [63, 64]. Further supporting its angiogenic role, silencing of AQP1 in ectopic endometrial tissues attenuated pathological angiogenesis, highlighting its potential relevance in endometriosis [65]. Moreover, AQP1 has been identified within the neovascular systems of atherosclerotic lesions in both human patients and murine models, suggesting that its expression correlates with vascular remodeling in atherosclerosis [66]. Elevated AQP1 immunoreactivity has also been observed in endothelial cells of blood vessels within inflamed periodontal and peri‐implant tissues, indicating a role in inflammatory angiogenesis [67]. Experimental interventions combining dexamethasone with gentamicin enema demonstrated suppressed angiogenesis in murine models of acute radiation proctitis, mediated by downregulation of both vascular endothelial growth factor (VEGF) and AQP1 [68]. Additionally, AQP1 may participate in myocardial angiogenic responses following ischemic injury, as suggested by recent experimental findings [69]. Studies using AQP1 knockout mice have shown that loss of AQP1 impairs angiogenesis, endothelial cell migration, and vascular integrity [20, 43], highlighting its essential function in vessel formation.

**FIGURE 3:**
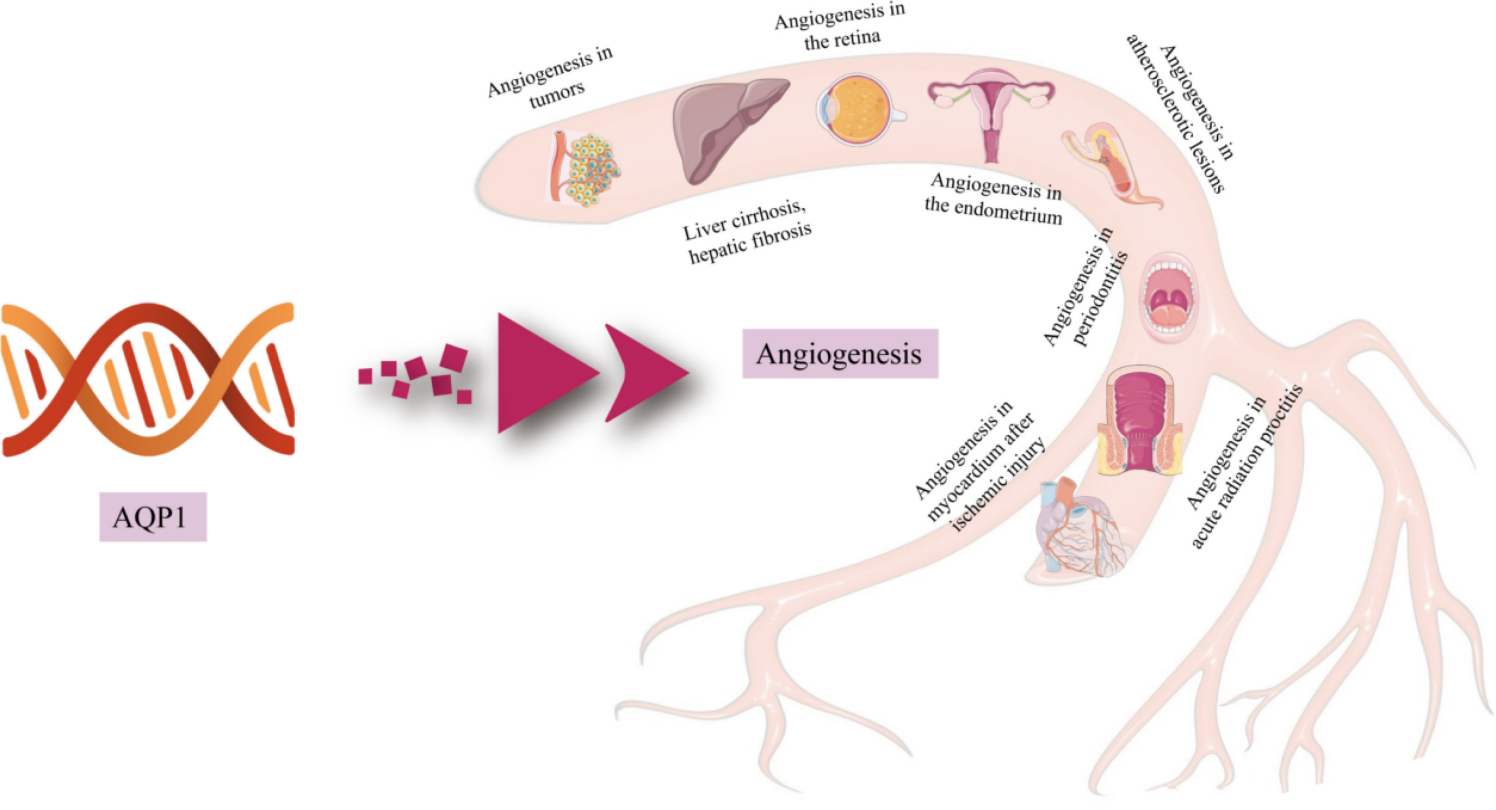
AQP1 is associated with pathological angiogenesis in various diseases.

In summary, AQP1 is aberrantly overexpressed in various disease conditions and has been shown to promote pathological angiogenesis in disorders such as cancer, liver fibrosis, diabetic retinopathy, and atherosclerosis, suggesting that AQP1 overexpression may be a key driver of disease‐associated neovascularization (Figure 3).

## 5 Potential Role of AQP1 in Vascular Neogenesis in DKD

In studies investigating the mechanisms underlying the progression of DKD, aberrant angiogenesis has been identified as one of the key driving factors in DKD progression [48, 57]. The proliferation of structurally and functionally immature neovessels leads to basement membrane thinning, endothelial cell swelling, and increased permeability, thereby causing dilation of glomerular capillaries, elevated permeability, and plasma albumin extravasation. These changes ultimately lead to albuminuria, which further exacerbates DKD progression [49, 70, 71, 72].

AQP1 is abundantly expressed in renal tissues and has been implicated in modulating key cellular processes such as migration, proliferation, and angiogenesis in multiple pathological conditions [59, 60, 61, 62, 63, 64, 65, 66, 67, 68, 69, 73, 74]. Notably, pathological angiogenesis observed in diabetic retinopathy, a microvascular complication closely related to DKD, has been linked to altered AQP1 expression [63]. This parallel underscores the potential translational relevance of targeting AQP1‐mediated angiogenic pathways to ameliorate DKD, a major microvascular complication of diabetes [47].

Zhang et al. [75] provided more direct evidence by demonstrating that AQP1 expression was markedly upregulated in the kidneys of spontaneous type 2 diabetic db/db mice and positively correlated with pathological angiogenesis, as evidenced by excessive activation of the VEGFA/VEGFR2 signaling pathway and increased CD31 expression. These findings were further validated in an in vitro model of mouse glomerular endothelial cells (MGECs) exposed to high glucose, in which elevated AQP1 levels were accompanied by enhanced endothelial migration, increased tube formation capacity, and upregulation of VEGFA/VEGFR2 and CD31. Treatment with hirudin reversed these pathological changes in a dose‐dependent manner in both models, resulting in downregulation of AQP1 expression, restoration of VEGFA/VEGFR2 signaling, and attenuation of endothelial activation and angiogenesis.

**FIGURE 4:**
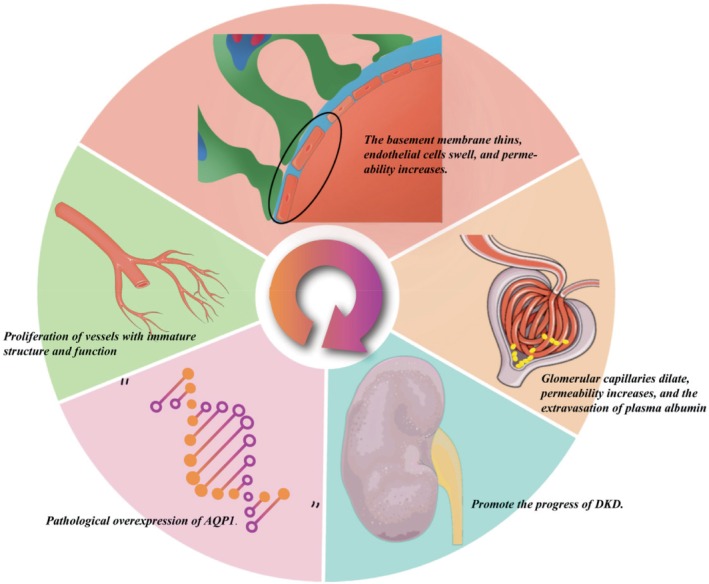
Potential role of AQP1 in vascular neogenesis in DKD.

Collectively, AQP1 serves not only as an important biomarker of vascular remodeling in DKD but also as a central regulator of endothelial activation and pathological angiogenesis [75]. Its aberrant overexpression can trigger abnormal neovascularization, leading to a cascade of downstream pathological events, including glomerular endothelial dysfunction, disruption of the glomerular filtration barrier, increased albuminuria, and further deterioration of renal function [49, 70, 71, 72], thereby highlighting the critical pathophysiological role of AQP1 in DKD progression (Figure 4). Targeted regulation of AQP1 expression may effectively suppress aberrant angiogenesis in DKD, offering an effective therapeutic strategy for DKD. Future research should focus on the signaling pathways involving AQP1 and the consequent downstream pathological alterations.

In addition, the development of therapeutic agents with higher specificity or multi‐target synergistic effects—especially through modern pharmacological screening of novel candidates capable of modulating AQP1 function—represents a key pathway to accelerate clinical translation. Certain natural products derived from traditional Chinese medicine have demonstrated potential in regulating AQP1 expression.

## 6 AQP1, Traditional Chinese Medicine (TCM), and Natural Products

In recent years, the regulatory effects of TCM and various natural compounds on AQP1 expression and function have attracted considerable scientific interest. Several studies have reported that specific TCM formulations and bioactive natural products possess the ability to modulate AQP1 levels, thereby influencing pathological processes such as angiogenesis, inflammation, and fluid balance. For instance, Xiaohong Wei et al. [76] demonstrated that the XinLi formula (XLF), a clinically utilized TCM prescription for treating chronic heart failure, significantly inhibited AQP1 expression and disrupted the interaction between angiotensin II receptor type 1 (AGTR1) and AQP1. Similarly, Yunhui Hu et al. [77] discovered that Compound Danshen Dripping Pills can regulate the expression of AQP1, among others. Atractylodes macrocephala Koidz (AMK) and *Paeonia lactiflora* Pall (PLP) have been found to reduce the levels of AQP1 and other related proteins [78]. Haitao Tu et al. [79] found that quercetin can regulate both AQP1 and AQP2, thereby reducing water retention and toxin accumulation. Additionally, compounds such as emodin, tanshinone IIA, and sennoside A have also been shown to be involved in the regulation of AQP1 [80, 81, 82]. While these findings are promising, it is important to emphasize that most studies remain preliminary and often lack comprehensive mechanistic elucidation or rigorous safety and efficacy evaluations. To translate these natural compounds and TCM formulations into effective therapeutic agents targeting AQP1 in DKD or other angiogenesis‐related conditions, extensive further research is necessary. This includes detailed molecular mechanism studies, standardized quality control of herbal preparations, pharmacokinetic and toxicological assessments, and ultimately, well‐designed clinical trials.

## 7 Conclusion

Current evidence indicates that AQP1 is a key driver of DKD pathogenesis and progression, primarily by promoting pathological angiogenesis in the renal microenvironment. However, the precise molecular mechanisms by which it regulates endothelial behavior, vascular permeability, and glomerular remodeling remain unclear. Future studies should focus on clarifying the signaling pathways involving AQP1 in DKD and their downstream pathological effects, exploring its cross‐talk with key angiogenic factors such as VEGFA/VEGFR2, and identifying safe, highly specific agents that can modulate AQP1 activity.

## Author Contributions

**Fengyi Zhang:** conceptualization, data curation, formal analysis, writing – original draft. **Jiayi Zhang:** formal analysis, writing – review and editing. **Xin Wang**, **Yaxin Chen**, **Jingjing Pan:** data curation, investigation. **Ziyang Cheng**, **Yufeng Zhang:** formal analysis. **Yujie Li:** funding acquisition, project administration, supervision, corresponding author. **Wenbo Wang:** project administration, supervision, writing – review and editing, corresponding author. **Linhua Zhao:** project administration, supervision, writing – review and editing, corresponding author.

## Disclosure

The authors have nothing to report.

## Ethics Statement

The authors have nothing to report.

## Conflicts of Interest

The authors declare no conflicts of interest.

## Data Availability

All relevant data can be found in this article.
